# Anti-proliferation and anti-migration effects of an aqueous extract of *Cinnamomi ramulus* on MH7A rheumatoid arthritis-derived fibroblast-like synoviocytes through induction of apoptosis, cell arrest and suppression of matrix metalloproteinase

**DOI:** 10.1080/13880209.2020.1810287

**Published:** 2020-09-02

**Authors:** Jia Liu, Qing Zhang, Ruo-Lan Li, Shu-Jun Wei, Yong-Xiang Gao, Li Ai, Chun-Jie Wu, Xu-Feng Pu

**Affiliations:** a School of Pharmacy, Chengdu University of Traditional Chinese Medicine, Chengdu, P.R. China; b Chengdu Institute for Food and Drug Control, Chengdu, P.R. China; c School of Basic Medicine, Chengdu University of Traditional Chinese Medicine, Chengdu, P.R. China; d School of Ethnic Medicine, Chengdu University of Traditional Chinese Medicine, Chengdu, P.R. China; e Chengdu Medical and Health Investment Group Co. Ltd, Chengdu, P.R. China

**Keywords:** Chinese medicine, anti-rheumatoid arthritis, synovial fibroblasts, molecular docking

## Abstract

**Context:**

*Cinnamomi ramulus,* the dry twig of *Cinnamomum cassia* Presl. (Lauraceae), has been reported to exert several activities such as antitumor, anti-inflammatory, and analgesic effects.

**Objective:**

This study investigates the effects of an aqueous extract of *Cinnamomi ramulus* (ACR) on rheumatoid arthritis (RA).

**Materials and methods:**

TNF-α-induced RA-derived fibroblast-like synoviocyte MH7A cells were incubated with ACR (0.1–2 mg/mL) for 24 h. The proliferation was tested using CCK-8 and colony formation assays. The migration and invasion abilities were measured using transwell tests and wound healing assays. Apoptosis and cell cycle were examined by flow cytometry. The potential mechanisms were determined by western blotting and quantitative real-time PCR. UPLC-QE-MS/MS was used for chromatographic analysis of ACR and its compounds were identified. Molecular docking strategy was used to screen the potential anti-RA active compounds of ACR.

**Results:**

We found that ACR induced apoptosis in MH7A cells at concentrations of 0.4, 0.8, and 1.2 mg/mL. The proliferation of MH7A cells was reduced and the cell cycle was blocked in the G2/M phase at concentrations of 0.2, 0.4, 0.6 mg/mL. Migration and invasion of MH7A cells were reduced through inhibiting the expression of MMP-1, MMP-2, and MMP-3. The molecular docking strategy results showed that 9 compounds in ACR have good affinity with protein crystal, and benzyl cinnamate (10–100 µg/mL) could inhibit cell migration and induce apoptosis.

**Conclusions:**

The anti-RA effect of ACR may be attributed to its anti-proliferative and anti-migration effects on synovial fibroblasts. These data suggest that *Cinnamomi ramulus* may have therapeutic value for the treatment of RA.

## Introduction

Rheumatoid arthritis (RA) is a chronic autoimmune joint disease characterized by synovial tissue inflammation, articular cartilage destruction, and joints malformation, whose exact cause is still not completely known (Müller-Ladner et al. 2005; Scott and Steer 2007). Synovial fibroblasts (SFs) play an important role in cartilage destruction by mediating most of the relevant pathways. Increasing evidence has suggested that activated SFs can manifest similar properties of tumour-like cells, such as hyperproliferation and insufficient apoptosis (Bustamante et al. 2017). The uncontrolled proliferation of SFs has been thought to contribute to the formation of RA (Huber et al. 2006). Meanwhile, SFs can also spontaneously secrete numerous pro-inflammatory cytokines and matrix metalloproteinases (MMPs), which play an important role in progressive destruction of articular cartilage (Abeles and Pillinger 2006; Malemud 2017). Thus, promoting apoptosis and inhibiting proliferation of SFs are believed to exert potential therapeutic effects on RA.

*Cinnamomi ramulus,* the dry twig of *Cinnamomum cassia* Presl. (Lauraceae), known as ‘*Guizhi*’ in China and ‘*Geiji*’ in Korea, has traditionally been used to relieve chronic joint pain in patients with arthritis (Kim et al. 2017; Zhang, Peng, et al. 2019). It is reported that the extract of *Cinnamomi ramulus* has a variety of biological activities, including antitumor, anti-inflammatory and analgesic activities (Sun et al. 2016; Park et al. 2018). These bioactivities of *Cinnamomi ramulus* might be dependent on the presence of certain classes of compounds in this plant, such as phenylpropanoids, monoterpenoids, sesquiterpenoids, sterols, etc. (Liu et al. 2018). Although the therapeutic potential of *Cinnamomi ramulus* for inflammatory reactions has been investigated, its effects on RA are currently poorly understood. In previous studies, we found that the traditional Chinese recipe *guizhi-shaoyao-zhimu* decoction has a significant anti-arthritic effect (Zhang, Peng, et al. 2019), and *Cinnamomi ramulus* is one of the most important herbal medicines in this traditional Chinese medicine (TCM) formula. In addition, there are also many other anti-RA TCM formulas with *Cinnamomi ramulus* as the main component, such as *huangqi guizhi wuwu* decoction, *guizhi fuzi* decoction, and *chai hu guizhi ganjiang* decoction, etc. (Kang et al. 2019; Yang 2019; Zeng et al. 2019). Some modern studies have confirmed the anti-RA potential of *Cinnamomi ramulus*, but the detailed molecular mechanisms remain unclear.

In this paper, we assessed the anti-RA effects of ACR on RA-derived fibroblast-like synoviocyte MH7A cells, focussing on possible mechanisms associated with suppressing proliferation, invasion and migration of MH7A cells, and inducing cell cycle arrest and apoptosis. In addition, we used the molecular docking to study the interactions between the main compounds of ACR and the RA-associated protein targets. And the influence of an important active component isolated from ACR on cell migration and apoptosis was discussed. Therefore, our present study provides a direction for anti-RA mechanism and drug research, as well as a reference for new treatment strategies of RA and the clinical application and development of *Cinnamomi ramulus*.

## Materials and methods

### Reagents and chemicals

Foetal bovine serum (FBS), phosphate buffered saline (PBS), penicillin-streptomycin, trypsin-EDTA and Dulbecco’s modified Eagle medium (DMEM) were purchased from GIBCO (Grand Island, NY, USA). The human TNF-α (tumour necrosis factor-α) was purchased from Pepro Tech (Rocky Hill, NJ, USA). Griess reagent, dimethyl sulfoxide (DMSO), Cell Counting Kit-8 (CCK-8 kit), BCA Protein Assay Kit, and Annexin V-FITC/PI apoptosis kits were purchased from the BOSTER Biol.Tech. Co. (Wuhan, China). Cell Cycle Staining kits were purchased from Beyotime Biotechnology Company (Haimen, China). TRIzol™ Plus RNA Purification Kit (Invitrogen life technology, Carlsbad, CA, USA), ReverTra Ace® qPCR RT Master Mix (Beans Biol Tech. Co., Tokyo, Japan) and SYBR Green RT-PCR reaction kit (QPK-201, Toyobo, Tokyo, Japan) were used in RT-PCR experiment. Benzyl cinnamate was purchased from the Desite Biology Co., Ltd., (Chengdu, China).

### Cell culture

Human synovial cell line MH7A and normal human fibroblast-like synoviocytes (HFLS) were purchased from the Beina Biological Company (Beijing, China), and cultured in DMEM medium with 10% FBS at 37 °C in 5% CO_2_ humidified atmosphere. Cells were passaged every 3–4 days, and cells obtained from the 5th to 10th passages were used for the experimental procedures.

### *Preparation of an aqueous extract of* Cinnamomi ramulus *(ACR)*

For laboratory study, authentic plant materials were purchased from the Neautus Chinese Herbal Pieces Ltd. Co. (Chengdu, China) on May 18, 2019, and were identified by Prof. Chun-Jie Wu (School of Pharmacy, Chengdu University of Traditional Chinese Medicine). A voucher specimen (CD123) has been deposited at Pharmacognosy Laboratory at Chengdu University of Traditional Chinese Medicine, China. Plant materials (200 g) were air-dried and cut into small pieces. The prepared samples were soaked by 300 mL of distilled water for 2 h and then left to boil for 1 h in a closed flask. Afterwards, the filtrates were cooled to room temperature and subsequently freeze-dried (−20 °C) to obtain the dry extract (0.6678 g). Then, the lyophilized powder of ACR was dissolved in DMEM at the appropriate concentrations for further experiments.

### Cell viability assay

Cell viability was determined using the CCK-8 kit according to the instruction of manufacturer. Briefly, MH7A cells were seeded at 5 × 10^3^ cells/well in 96-well culture plates in DMEM containing 10% FBS, incubated overnight. Then cells were stimulated with TNF-α (20 ng/mL) and exposed at various concentrations of ACR for 24 h. After that, CCK-8 was added to each well of the plate and incubated at 37 °C for 1.5 h. The resulting optical density was detected at 450 nm by a multi-detection iMARK microplate reader (BIO-RAD, Hercules, CA, USA).

### Apoptosis assay

MH7A cells (5 × 10^5^ cells/well) were seeded onto 6-well plates and cultured for 12 h. After the incubation, cells were treated with different concentrations ACR (0.4, 0.8, 1.2 mg/mL) and TNF-α (20 ng/mL) for 24 h. After treatment, the cells were harvested and washed with PBS, and subsequently stained with AnnexinV FITC/PI apoptosis assay kit according to the manufacturer’s instructions. Afterward, apoptosis was analysed by laser scanning confocal microscope (Leica TCS SP8 STED, Heidelberg, Germany) and FACS Calibur flow cytometry (Becton Dickinson, San Jose, CA, USA).

### Cell cycle assay

MH7A cells (5 × 10^5^ cells/well) were inoculated on 6-well plates and cultured for 12 h. Cells were incubated with different concentrations of ACR (0.2, 0.4, 0.6 mg/mL) and TNF-α (20 ng/mL) for 24 h. For analysis of the cell cycle distribution, the supernatant was discarded, and attached cells were harvested and fixed in cold 75% ethanol. The cells were then kept at −20 °C for 24 h before analysis. Cells were stained with propidium iodide (PI; Sigmar), and the DNA content was determined by FACS Calibur flow cytometry (Becton Dickinson, San Jose, CA, USA) to analyse the cell cycle.

### Scratch wound healing experiment

Cell migration assay was performed by the scratch wound healing assay. MH7A cells (5 × 10^5^ cells/well) were seeded into a 6-well plate, and when the cells covered the whole bottom surface of the well, the serum-free medium was used to continue the culture for 12 h, so as to eliminate the influence of normal cell growth on the scratch experimental results. After that, a p200 pipet tip was used to make a scratch at the bottom of the well, and the cells were treated with TNF-α (20 ng/mL) and ACR (0.2, 0.4 and 0.6 mg/mL). After 24 h of scratch, cells were stained with crystal violet, and an inverted microscope (Nikon TS2, Tokyo, Japan) was used to observe and photograph the scratch area, and then the distance change of the scratch area was measured and analysed.

### Transwell experiment

Transwell chamber was used to assess the migration and invasion capacity of cells. The cells (1 × 10^5^ cells/well) were seeded in a serum-free medium in the upper chamber of transwell (Corning Inc, Corning, NY, USA), and a DMEM medium containing 20% serum in the lower chamber, and cells treated with TNF-α (20 ng/mL) or ACR (0.2, 0.4 and 0.6 mg/mL) for 24 h. After that, the transwell chambers were removed, and the cells inside the membrane were gently scraped off with a cotton swab, while the cells outside the membrane were thought to be migratory or invasive. The migratory or invasive cells were fixed with 4% paraformaldehyde at room temperature for 20 min, washed with PBS three times, and stained with crystal violet for 10 min. Finally, an inverted microscope (Nikon TS2, Tokyo, Japan) was used to observe and photograph. Image J software (version 1.51, NIH, MD) was used to count invasive cells. In contrast to the migration experiment, 0.1% matrigel (BD) was spread at the bottom of the transwell chamber in the invasion experiment.

### Colony formation assay

MH7A cells (1 × 10^3^ cell/well) were seeded in 6-well plates and treated with TNF-α (20 ng/mL) and ACR (0.2, 0.4 and 0.6 mg/mL) for 24 h. The cells were cultured in a new medium for a week. Then the cells were immobilized and stained with crystal violet, and the number of colony formation was counted in a random microscopic field and the photos were taken.

### Western blot analysis (WB)

MH7A cells (5 × 10^5^ cell/well) were treated with TNF-α (20 ng/mL) and various concentrations of ACR (0.2, 0.4, 0.6 mg/mL) for 24 h. RIPA lysis buffer (containing protease and phosphatase inhibitors) was used to collect protein from cell samples. The supernatant of lysate was boiled, and total protein was measured using a BCA kit. Proteins were separated by SDS-PAGE, and then transferred to polyvinylidene fluoride (PVDF) membrane. The membranes were blocked with 5% BSA (Bovine SerumAlbumin) in TBST (Tris-Buffered Saline and Tween 20) at room temperature for 1 h, followed by exposure to the corresponding primary antibodies overnight at 4 °C. After washing with TBST for three times, the membranes were incubated with the secondary antibodies. Proteins were scanned using the ECL detection system, and the gel images were analysed using the Image J (v1.51) image processing software.

### RNA extraction and quantitative real-time PCR (qPCR)

According to the manufacturer’s instructions, total RNA was extracted using Trizol reagent, and each sample was reverse transcribed using the cDNA synthesis kit. The mRNA expression of MMP-1, 2 and 3, P53, P21, CDK4 and cyclin D were determined by qRT-PCR assays using SYBR Green PCR Premix Ex Taq II reagents on a Light Cycler 480 II real-time system (Roche, Mannheim, Germany). GAPDH, a house-keeping gene, was used for was used as a quantitative control for RNA levels. Relative gene expression was calculated by the ^ΔΔ^Ct method. The sequences for the relevant primers are listed in Table 1. qPCR was run with an initial denaturation step at 95 °C for 30 s, followed by extension step at 57 °C and 30 s for 44 cycles.

**Table 1.** Primer sequences used for quantitative real-time PCR.

Target genes	Sequence	
MMP-1	Forward: Reverse:	CTCAATTTCACTTCTGTTTTCTG CATCTCTGTCGGCAAATTCGT
MMP-2	Forward: Reverse:	CGGTGCCCAAGAATAGATG AAAGGAGAAGAGCCTGAAGTG
MMP-3	Forward: Reverse:	GGCTTCAGTACCTTCCCAGG GCAGCAACCAGGAATAGGTT
p53	Forward: Reverse:	CAGCCAAGTCTGTGACTTGCACGTAC CTATGTCGAAAAGTGTTTCTGTCATC
p21	Forward: Reverse:	AGATGTCCAGCCAGCTGCACCTGAC CTATGTCGAAAGTGTTTCTGTCATC
CDK4	Forward: Reverse:	ACGCCTGTGGTGGTTACG CCATCTCTGGCACCACTGAC
Cyclin D	Forward: Reverse:	CAAACAGATCATCCGCAAAC GCGTGTGAGGCGGTAGT
GAPDH	Forward: Reverse:	AGCCACATCGCTCAGACAC GCCCAATACGACCAAATCC

### Qualitative UPLC-QE-MS/MS analysis

For qualitative analysis, a Thermo Scientific Q Exactive Orbitrap HRMS (Thermo Fisher Scientific, Massachusetts, USA) was connected to a Thermo Scientific Vanquish UPLC (Thermo Fisher Scientific, Massachusetts, USA). Chromatographic separation was achieved on a Thermo Scientific™ Accucore™ C18 (3 × 100 mm, 2.6 μm) in a thermostatically controlled column compartment (30 °C). The aqueous and organic mobile phases used were 0.1% formic acid in water (A) and methyl alcohol (B), respectively. A gradient elution system was set up as follows: 0–20 min, 5–80% B; 20–30 min, 80–95% B; 30.1–35 min, 95–5% B. The flow rate was 0.3 mL/min, and 2 μL of the extraction was injected to the LC system. The instrument was operated in positive ion mode to perform full-scan analysis over an *m/z* range of 100–1500. And the optimized parameters were set as follows: the sheath gas flow rate – 35 L/min; spray voltage – 3000 V; capillary temperature – 320 V; aux gas flow rate – 10.00 L/min; max spray current – 100A; probe heater temperature – 350 °C; S-lens RF level – 50.00%.

### Molecular docking studies

According to the preliminary analysis by UPLC-QE-MS/MS, ACR contained 9 chemical components. We found the structure formulas of these compounds in PubChem (https://pubchem.ncbi.nlm.nih.gov/), and used ChemDraw software (version 14.0, CambridgeSoft, Cambridge, MA, USA) to draw the structures of these compounds and save them as MOL files. Then, according to the previous WB and q-PCR results, Bcl-2, caspase 3, MMP3, CDC2, cyclin B1, CDK4, P21, P53 and cyclin D were selected as the functional targets of RA. Human protein complex crystals with prototype small molecular ligands for these targets were downloaded from the PDB (http://www.rcsb.org) website, and the standard names of selected targets were confirmed by Uniprot (http://www.uniprot.org/). Molecular docking was performed using Discovery studio software (DS, version 4.5.0, Biovea Inc., Omaha, NE, USA). In simple terms, after importing the target protein crystal composite and the MOL files of compounds into DS software, Ligand Docking module and molecular Docking of rigid Docking were used according to prototype Ligand binding site. Compounds with higher docking scores than the prototype ligand were considered as the active compounds capable of binding to the target.

### Effects of benzyl cinnamate on cell migration and apoptosis in synovial fibroblasts

In order to explore the effects of compound **9** (benzyl cinnamate) on cell migration and apoptosis in synovial fibroblasts, MH7A cells were treated with TNF-α (20 ng/mL) and different concentrations benzyl cinnamate (10–100 μg/mL) for 24 h, and the cell viability was evaluated by CCK-8. Based on the above experimental methods, after MH7A cells were treated with TNF-α (20 ng/mL) and benzyl cinnamate (10, 20, 40 μg/mL) for 24 h, the cell migration was determined by the wound healing assay and apoptosis was tested by flow cytometry.

### Statistical analysis

All results were presented as mean ± standard deviation (SD). Statistical significance between groups was analysed by Student’s *t*-test or ANOVA of SPSS software (version 19). Statistical significance was defined as a *p* < 0.05.

## Results

### ACR inhibits the proliferation of synovial fibroblasts

**Figure 1.**
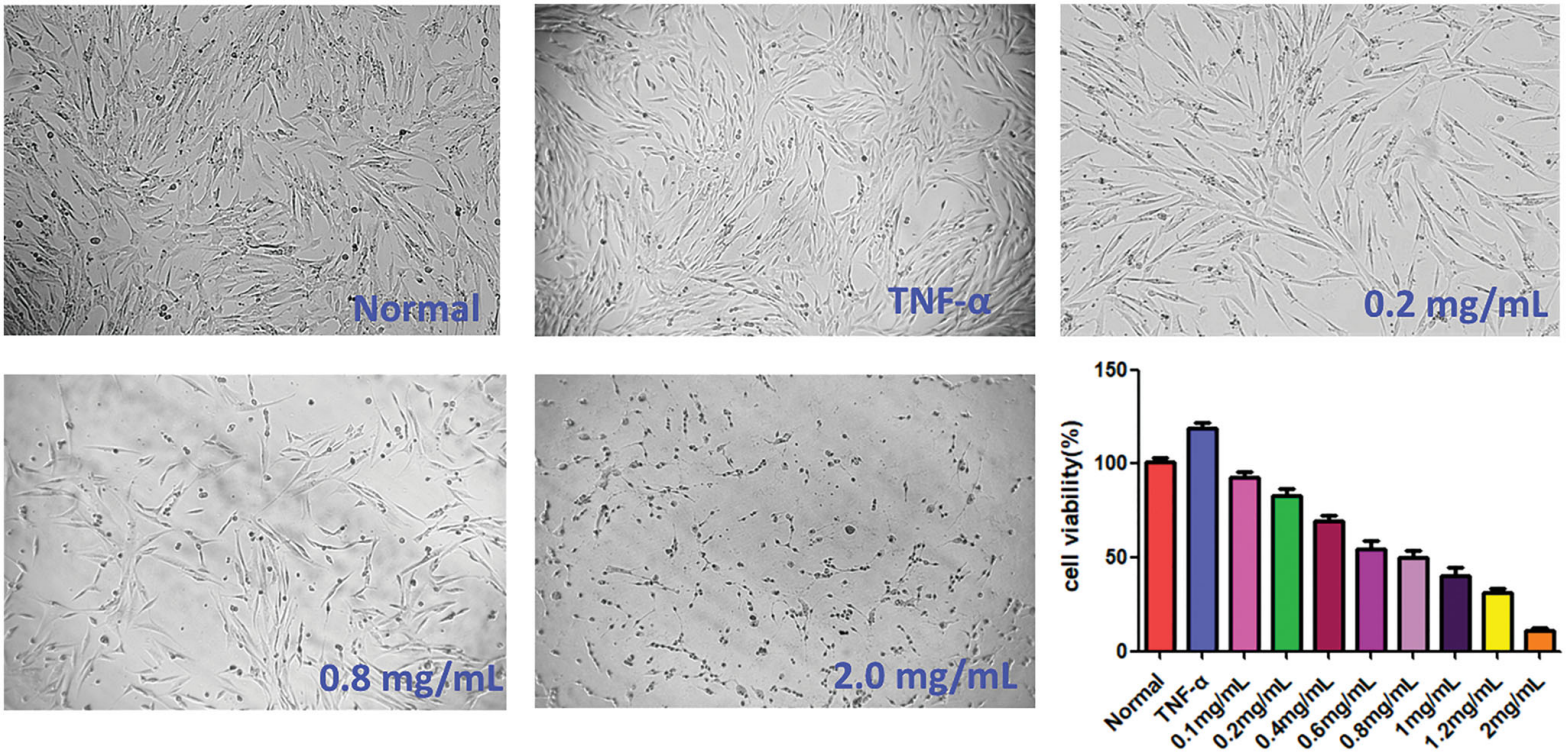
Cytotoxicity of ACR on MH7A cells. MH7A cells were treated with different concentrations of ACR (0.1, 0.2, 0.4, 0.8, 1.0 and 2.0 mg/mL) for 24 h, and cell viability was measured by CCK-8 assay. ACR: an aqueous extract of *Cinnamomi ramulus*.

CCK-8 assay was carried out to examine the cytotoxicity of ACR (0.1, 0.2, 0.4, 0.8, 1.0 and 2.0 mg/mL) on TNF-α stimulated MH7A cells. As shown in Figure 1, it revealed that ACR could inhibit the proliferation of MH7A cells in a concentration-dependent manner. Interestingly, ACR had no obvious effect on HFLS cells viability at the same concentration (Supplementary Figure S1). Therefore, the ACR concentration (0.2–1.2 mg/mL) used in this experiment was suitable for the *in vitro* studies of MH7A RA-derived fibroblast-like synoviocytes.

**Figure 2.**
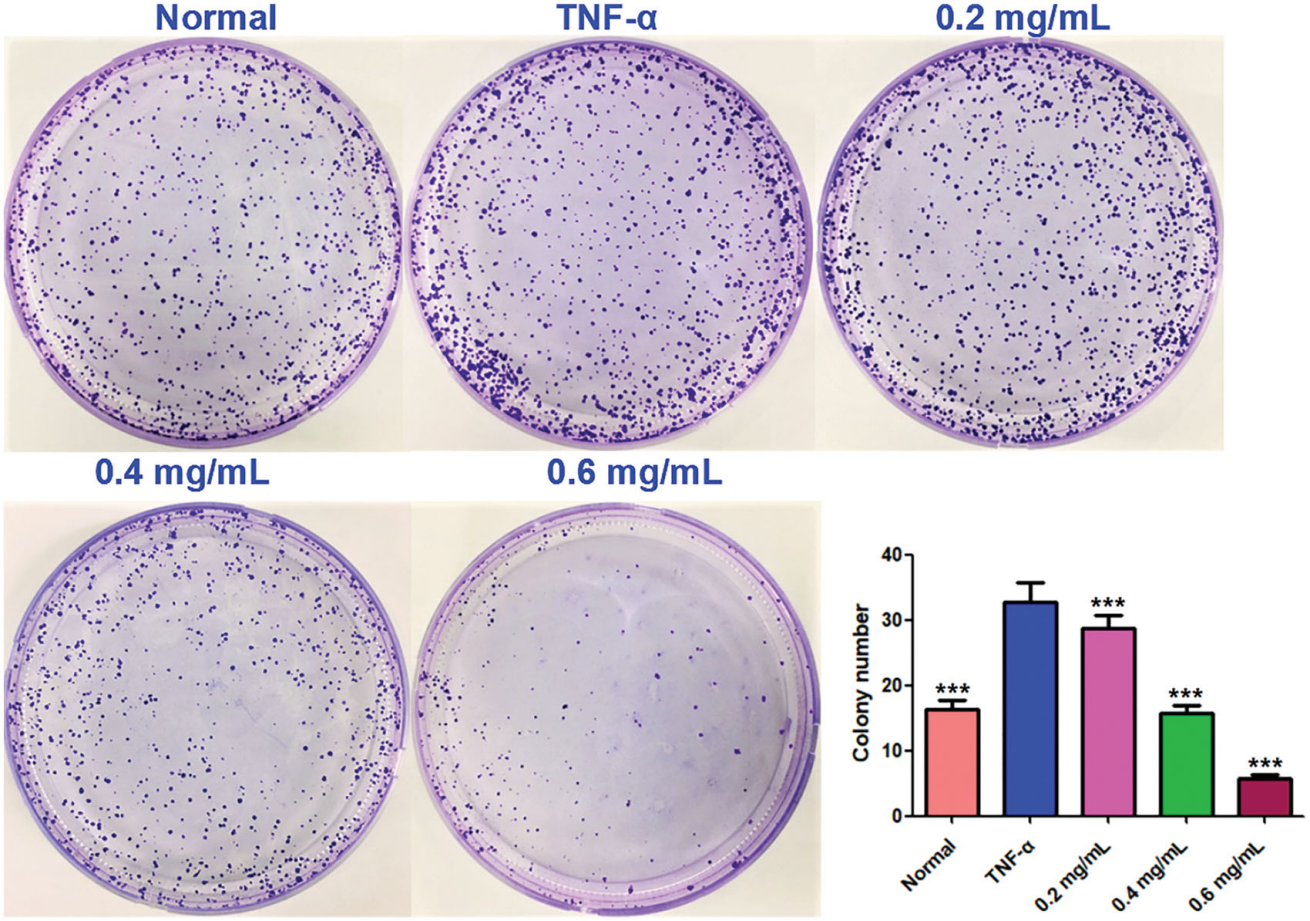
Colony formation assays. Equal numbers of MH7A cells were plated and treated with various concentrations of ACR for 10 days. The number of colony formation was counted in a random microscopic field. Data are expressed as mean ± SD (n = 3), ***p < 0.001, vs. TNF-α group. ACR: an aqueous extract of *Cinnamomi ramulus*.

Furthermore, colony formation is an *in vitro* cell survival assay based on the ability of a single cell to grow into a colony. The assay essentially tests every cell in the population for its ability to undergo ‘unlimited’ division, and to measure the proliferation capacity of the cells (Franken et al. 2006). Similar to the results of CCK-8 assay, colony formation assay showed that ACR (0.2, 0.4 and 0.6 mg/mL) significantly and concentration-dependently inhibited the colony formation rate (*p* < 0.001), compared to the TNF-α group (MH7A cells treated by TNF-α alone) (Figure 2).

### ACR induces apoptosis in synovial fibroblasts

**Figure 3.**
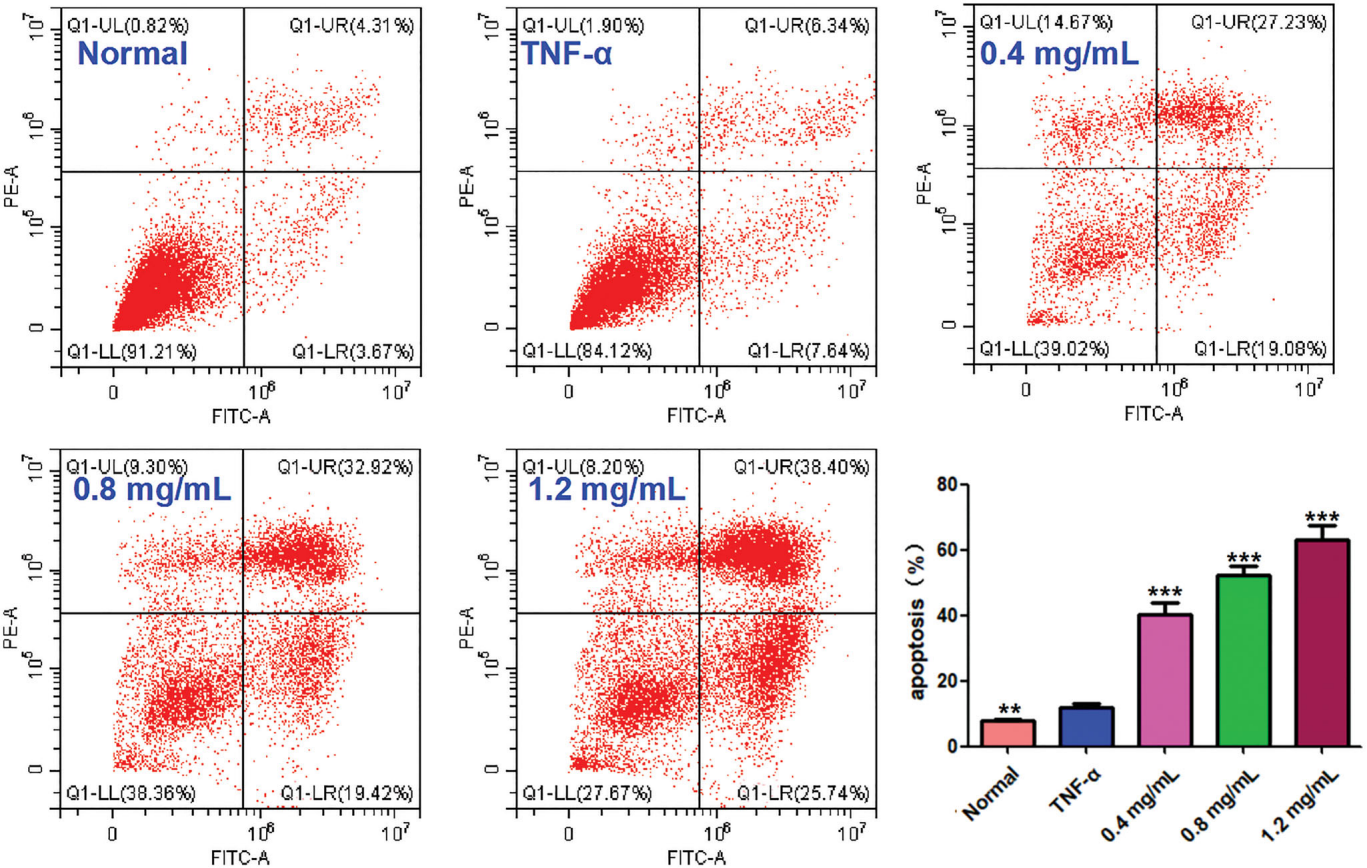
Effect of ACR on apoptosis of MH7A cells. MH7A cells were treated with ACR (0.4, 0.8, 1.2 mg/mL) for 24 h, followed by assessment of apoptosis by flow cytometric analysis. Data are expressed as mean ± SD (n = 3), **p < 0.01, ***p < 0.001, vs. TNF-α group. ACR: an aqueous extract of *Cinnamomi ramulus*.

**Figure 4.**
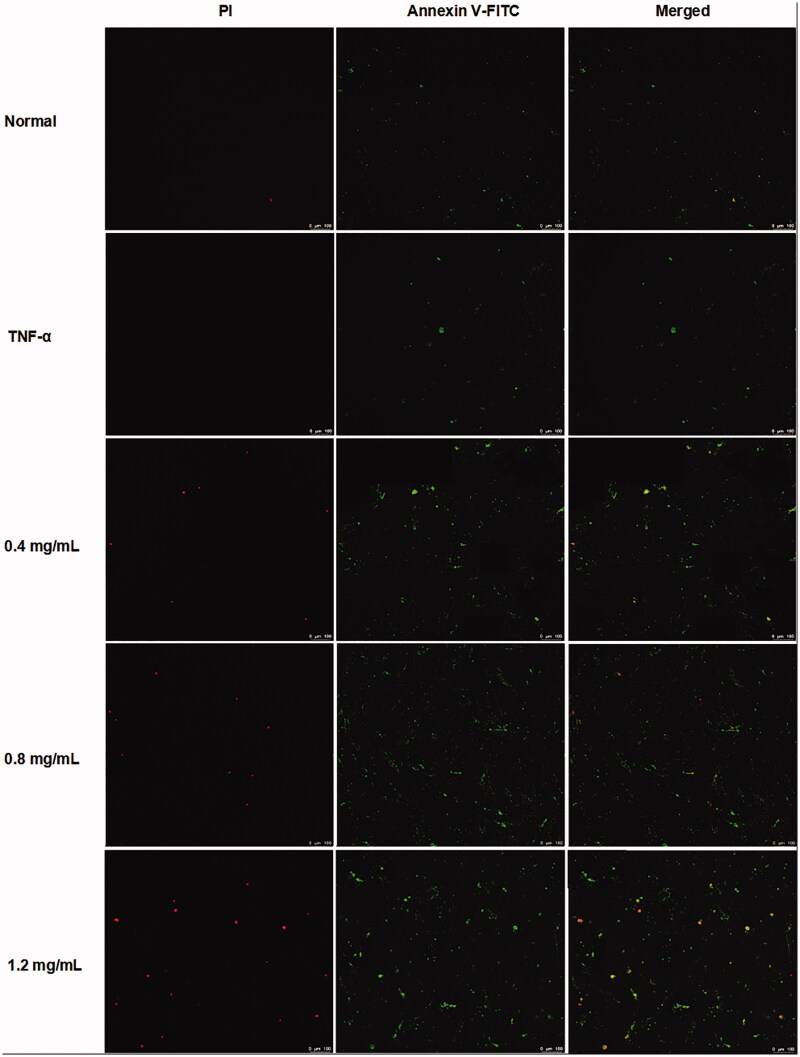
ACR induces apoptosis in MH7A cells. MH7A cells were treated with ACR (0.4, 0.8, 1.2 mg/mL) for 24 h, followed by assessment of apoptosis under laser scanning confocal microscope. ACR: an aqueous extract of *Cinnamomi ramulus*.

In order to determine whether the cytotoxic effects of ACR on MH7A cells are related to induction of apoptosis or not, flow cytometry and laser scanning confocal microscope with AnnexinV FITC/PI staining were used to determine the apoptosis in MH7A cells. As shown in Figure 3, different concentrations of ACR (0.4, 0.8, 1.2 mg/mL) could significantly induce the apoptosis of MH7A cells (*p* < 0.001), compared to the TNF-α group. Furthermore, similar to the results of flow cytometry analysis, the MH7A cells treated with ACR (0.4, 0.8, 1.2 mg/mL) were also observed using laser scanning confocal microscope to exhibit early and late apoptotic characteristics (Figure 4).

**Figure 5.**
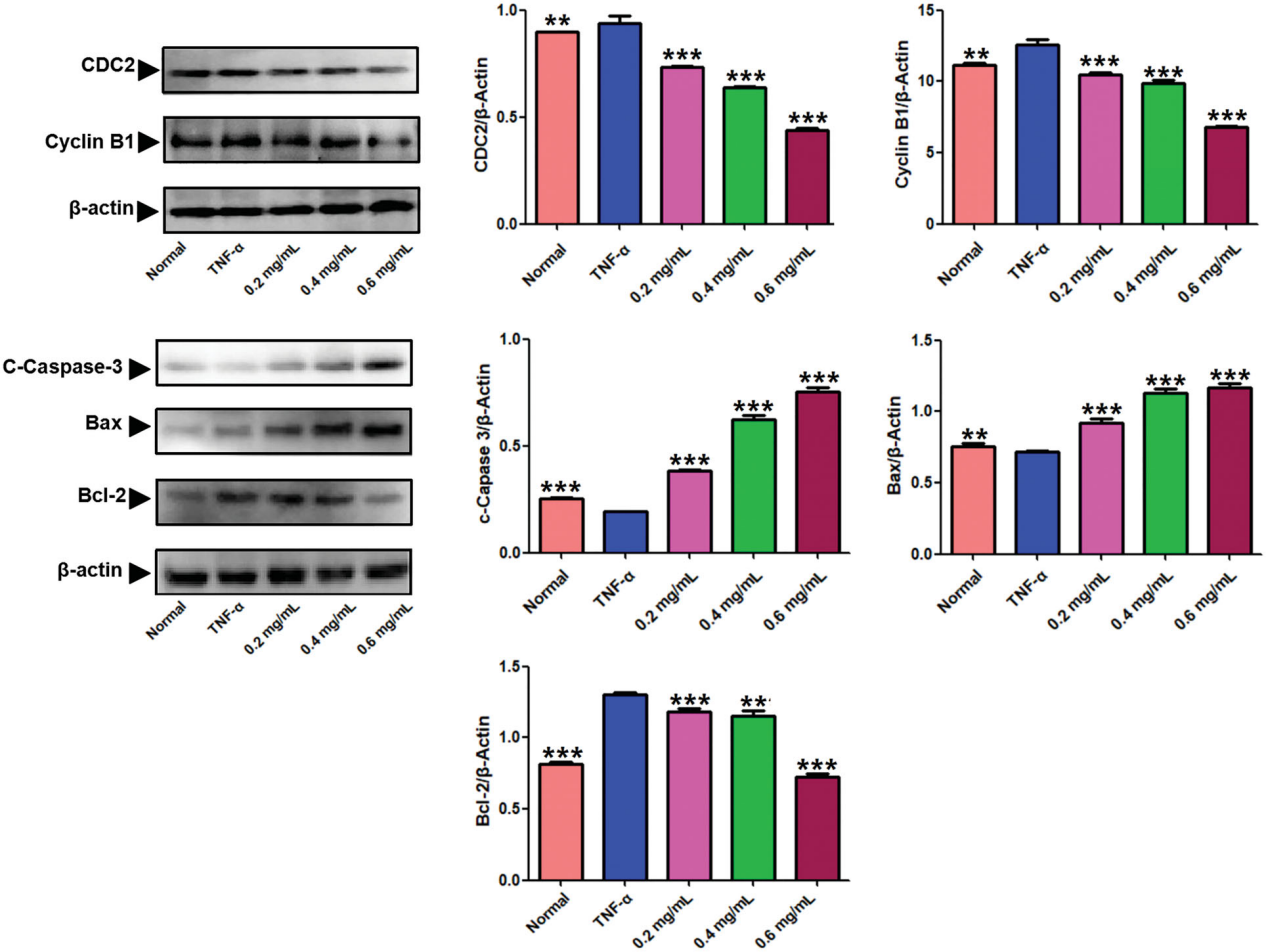
Results of the western blotting assays. Western blot analysis was applied to detect the protein level of C-caspase-3, Bax, Bcl-2, cyclin B1 and CDC2 in MH7A treated with ACR (0.2, 0.4, 0.6 mg/mL) for 24 h. β-Actin was used as the sample loading control, Data are expressed as mean ± SD (n = 3), **p < 0.01,***p < 0.001, vs. TNF-α group. ACR: an aqueous extract of *Cinnamomi ramulus*.

Furthermore, we analysed the effect of ACR on expression of apoptosis-associated proteins by western blotting assays. As shown in Figure 5, ACR (0.2, 0.4, 0.6 mg/mL) significantly elevated the expression of Cleaved (C)-caspase-3 (*p* < 0.001) and Bax (*p* < 0.001), whereas reduced the expressions of Bcl-2 (*p* < 0.001) in concentration-dependent manner compared to the TNF-α group, which was consistent with results from previous apoptosis analysis.

### ACR induces G2/M phase arrest in synovial fibroblasts

**Figure 6.**
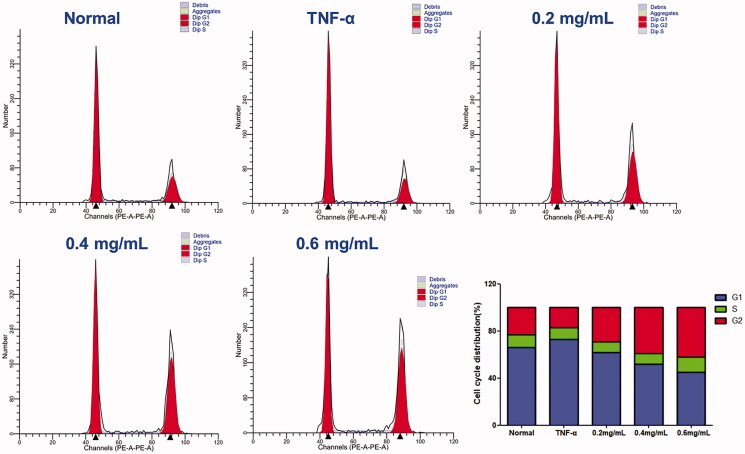
Effect of ACR on the cell cycle in MH7A cells. Flow cytometry was used to assess the cell cycle rate of MH7A cells treated with various concentrations of ACR (0.2, 0.4, 0.6 mg/mL). Histogram represented the statistical analysis of the relative expression level of G2/M related proteins. Data are expressed as mean ± SD (n = 3). ACR: an aqueous extract of *Cinnamomi ramulus*.

**Figure 7.**
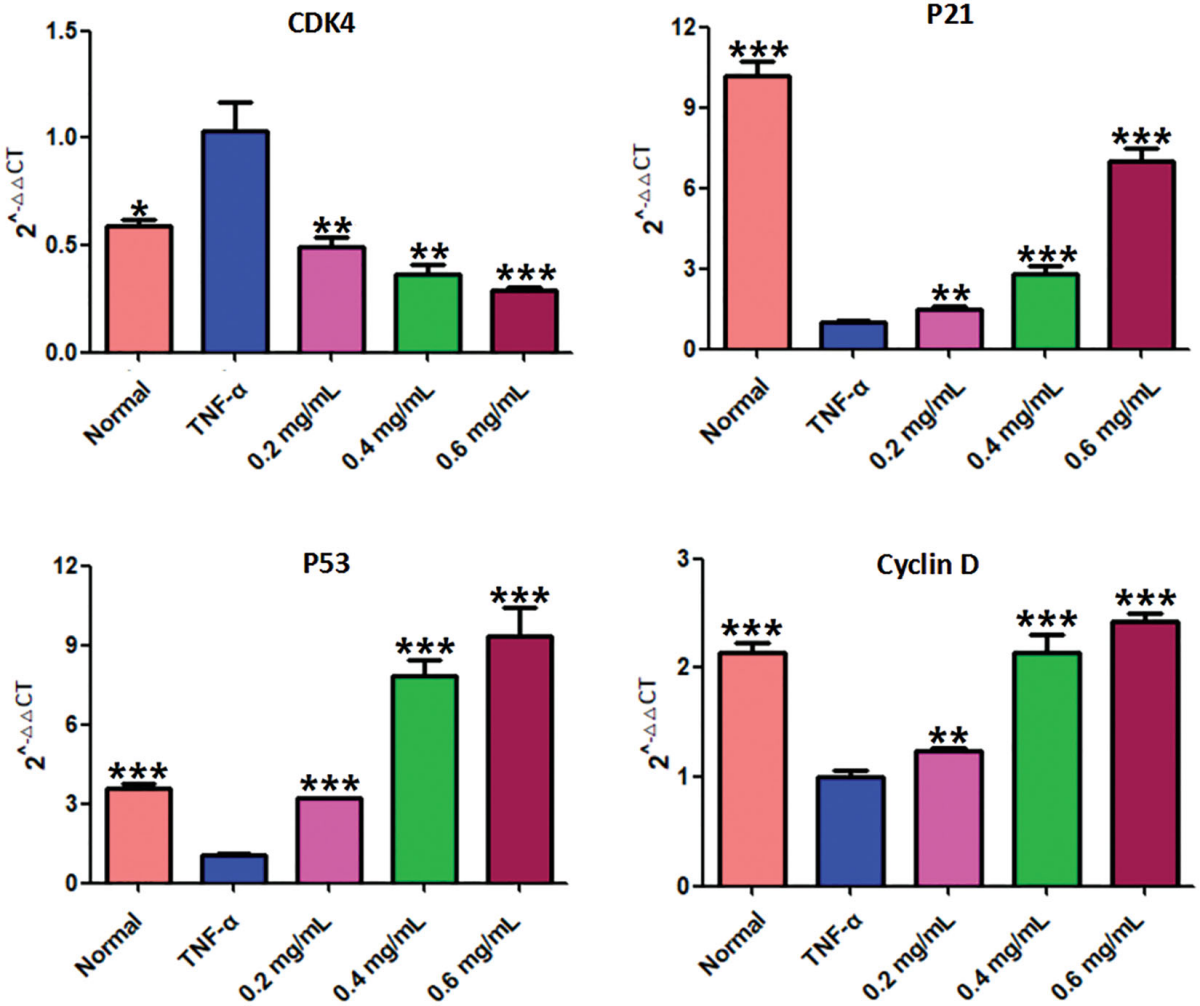
Results of the RT-PCR analysis on P53, P21, CDK4 and cyclin D. Quantitative real-time PCR was performed as described in the Materials and methods. Data are expressed as mean ± SD (n = 3), **p < 0.01, ***p < 0.001, vs. TNF-α group. ACR: an aqueous extract of *Cinnamomi ramulus*.

The effect of ACR on cell cycle distribution was studied by flow cytometric analysis. The results showed that ACR (0.2, 0.4, 0.6 mg/mL) treatments resulted in a concentration-dependent increase in the proportion of G2 phase cells, accompanied by a decrease in G1 phase cells (Figure 6). Therefore, in order to further investigate whether the cell cycle arrest due to the exposure to ACR or not, we examined the relative levels of cell cycle-related genes and proteins after treatment with different concentrations of ACR (0.2, 0.4, 0.6 mg/mL). Western blotting and q-PCR assays showed that compared with the TNF-α group, ACR treatment (0.2, 0.4, 0.6 mg/mL) resulted in the up-regulation of cyclin D (*p* < 0.01), P53 (*p* < 0.01) and P21 (*p* < 0.01), accompanied by the down-regulation of CDK4 (*p* < 0.01), cyclin B1(*p* < 0.01) and CDC2 (*p* < 0.01) (Figures 5 and 7). These results were consistent with the results of G2 cell cycle arrest obtained by flow cytometry, and confirming the involvement of ACR in the regulation of cell cycle.

### ACR inhibits cell migration and invasion in synovial fibroblasts

**Figure 8.**
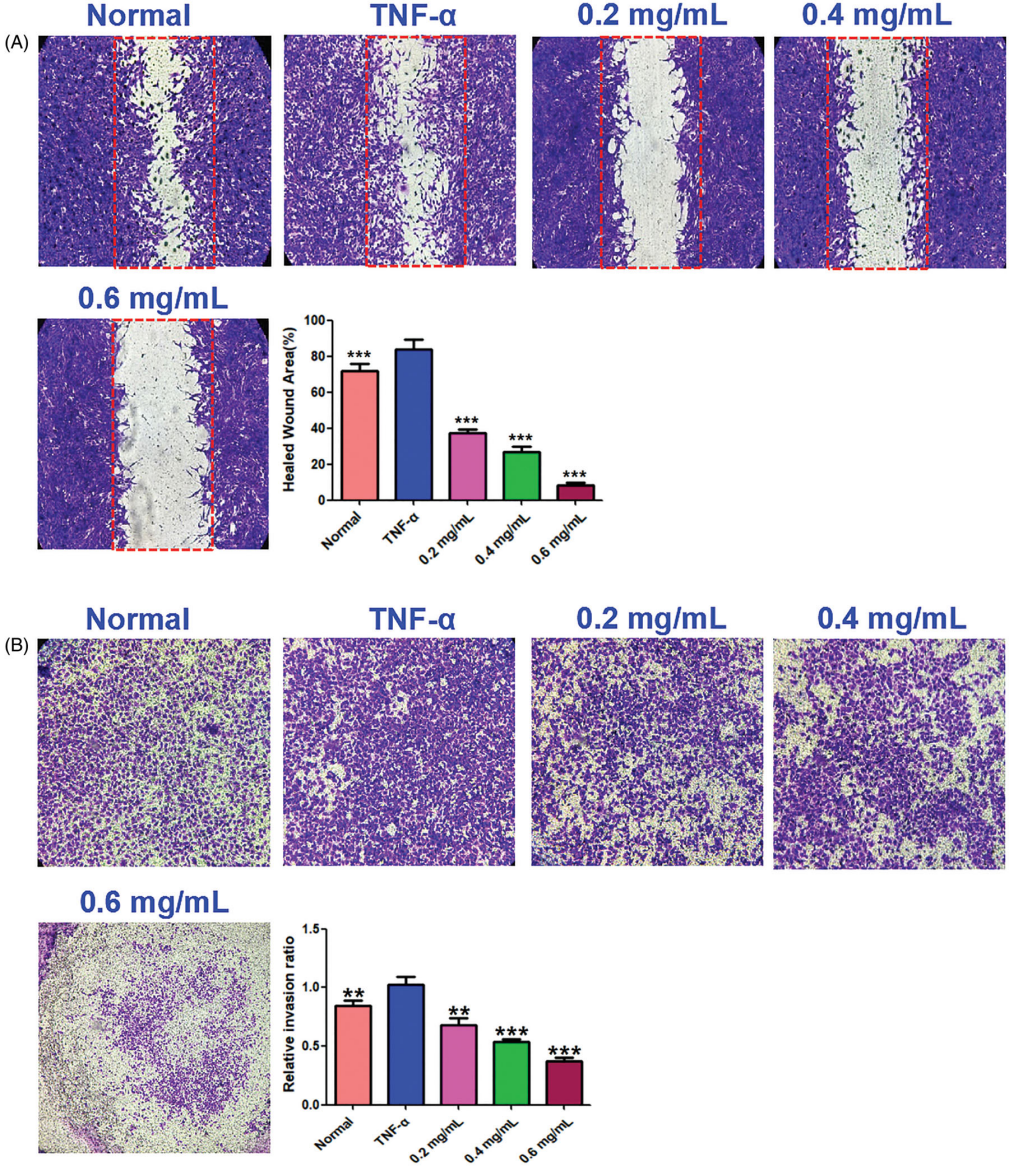
Effects of ACR on migration ability of MH7A cells in wound healing test (A) and transwell migration assay (B) of MH7A cells (200×). Data are expressed as mean ± SD (n = 3), **p < 0.01, ***p < 0.001, vs. TNF-α group. ACR: an aqueous extract of *Cinnamomi ramulus*.

**Figure 9.**
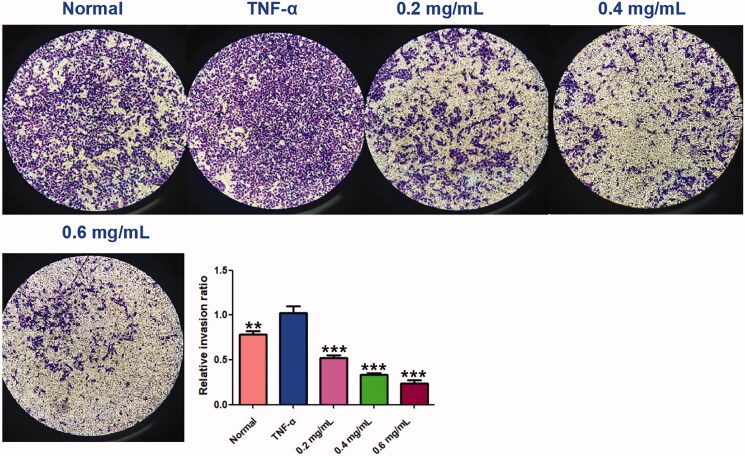
ACR suppresses the invasion ability of MH7A cells. Cell invasion abilities were detected by Matrigel Transwell assay. *Cinnamomi ramulus* reduced the invasion of MH7A cells in dose-depended manner as demonstrated by representative microscope graphs (200×). Data are expressed as mean ± SD (n = 3), **p < 0.01, ***p < 0.001, vs. TNF-α group. ACR: an aqueous extract of *Cinnamomi ramulus*.

The wound healing and transwell assays carried out to determine the cell migration and invasion in MH7A cells, and the results showed that ACR (0.2, 0.4, 0.6 mg/mL) can inhibit cell migration and invasion of MH7A cells with a concentration-dependent manner. In the wound healing assay (Figure 8(A)), we found that the scratch prepared by pipette tip in the normal and TNF-α groups were almost fully filled with MH7A cells, while the cells migratory ability in the ACR-treated group were decreased. Similar to the results of wound healing assay, significant decreased cells were detected in ACR (0.2, 0.4, 0.6 mg/mL) treated MH7A cells in the transwell migration experiment (*p* < 0.01), compared to the TNF-α groups (Figure 8(B)). Furthermore, transwell chambers with matrigel were used to detect the invasive capacity of MH7A cells. The results showed ACR (0.2, 0.4, 0.6 mg/mL) caused significant suppression of the invasive cells in the matrigel-transwell experiment (*p* < 0.001), compared to the TNF-α group (Figure 9).

**Figure 10.**
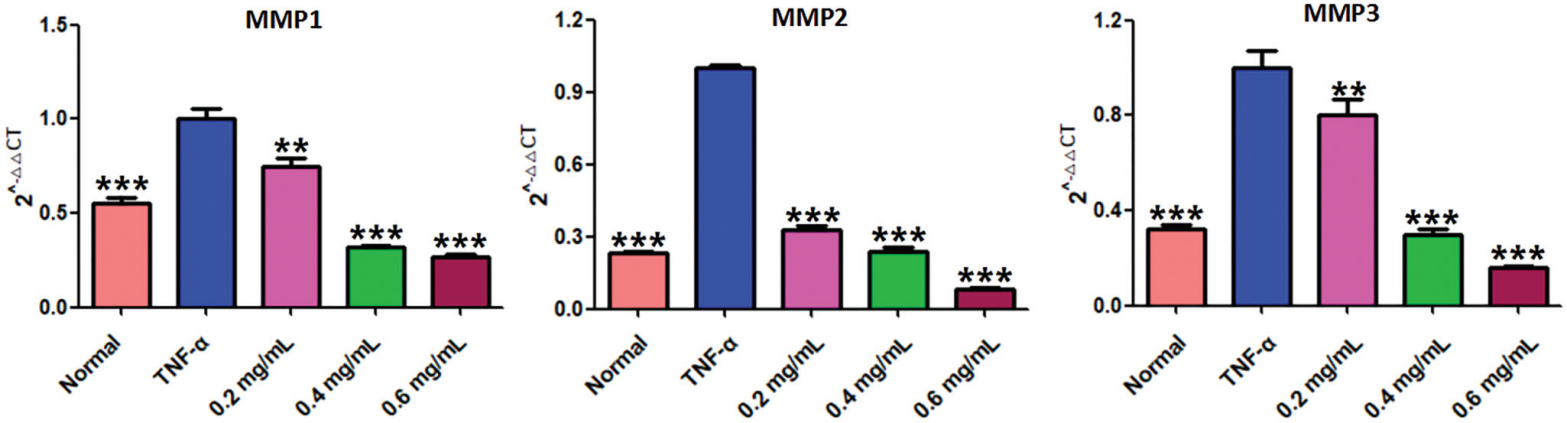
ACR suppresses the expression of MMPs. The mRNA levels of MMP-1, -2 and -3 were determined by using Quantitative real-time PCR (q-PCR). Data are expressed as mean ± SD (n = 3), **p < 0.01, ***p < 0.001, vs. TNF-α group. ACR: an aqueous extract of *Cinnamomi ramulus*.

Cell migration and invasion are closely related to matrix degradation, a process mainly dependent on the activities of degradation enzymes such as MMPs (Araki and Mimura 2017). The quantitative real-time PCR (q-PCR) assays of MMP-1, 2 and 3 were carried out to explore its related molecular mechanism. As shown in Figure 10, all testing MMPs (including MMP-1, MMP-2, MMP-3) were significantly increased by TNF-α stimulation (*p* < 0.001), compared to the normal MH7A cells. Interestingly, the increased MMPs could be markedly reduced by ACR (0.2, 0.4, 0.6 mg/mL) (*p* < 0.01) compared to the TNF-α group. The data mentioned above manifested that ACR could obviously reduce the migration and invasion of TNF-α stimulated MH7A cells via suppression of MMPs expression.

### Results of constituent analysis of ACR by UPLC-QE-MS/MS

**Figure 11.**
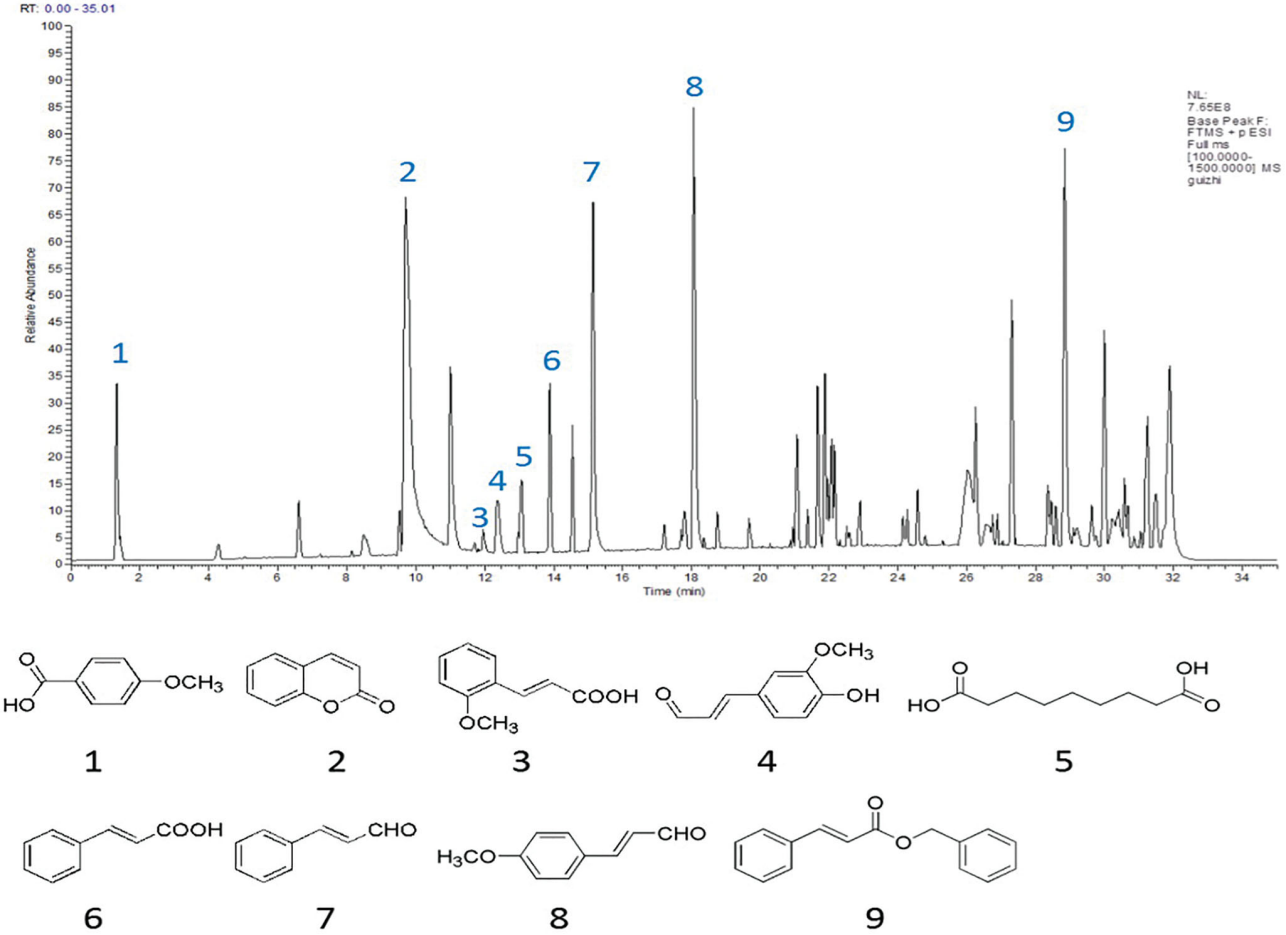
Result of the UPLC-QE-MS/MS assays of ACR. The MS total ion chromatograms (TIC) of ACR and chemical structures of identified compounds in ACR. ACR: an aqueous extract of *Cinnamomi ramulus*.

UPLC-QE-MS/MS was used to analyse the freeze-dried powder of ACR, as shown in the materials and methods section. Figure 11 showed the MS total ion chromatograms (TIC) provided by analysis of the ACR in positive ionization modes. To qualitatively investigate the main constituents of ACR, we confirmed the identity of the analyte by comparing individual retention times (*t*_R_), online MS spectra and reference standards in the literature. Peaks 1–9 were unequivocally identified as anisic acid, coumarin, 2-methoxycinnamic acid, coniferyl aldehyde, azelaic acid, cinnamic acid, cinnamaldehyde, 4-methoxy-cinnamaldehyde and benzyl cinnamate, respectively. Reference standards were used to confirm the retention times, accurate mass and fragment ions. Tentatively identified compounds in the ACR (Figure 11) and the main parameters supporting their identification are presented in Table 2.

**Table 2.** Chemical constituents identified from ACR.

Peak	*t*_R_ (min)	Measured mass (*m/z*)	MS/MS fragments (*m/z*)	Identification
1	1.32	153.05481 [M + H]^+^	135, 107, 92, 77	Anisic acid (Aliboni et al. 2011)
2	9.72	147.04434 [M + H]^+^	103, 91, 77, 65	Coumarin (Chen et al. 2016)
3	12.01	179.07014 [M + H]^+^	161, 137, 135	2-Methoxycinnamic acid (Yang 2017)
4	12.39	179.07024 [M + H]^+^	161, 147, 119, 107, 91, 79, 65	Coniferyl aldehyde (Yang 2017)
5	13.07	189.09706 [M + H]^+^	152, 124, 111, 98, 84, 83, 73, 69, 60, 55 , 41	Azelaic acid (He et al. 2002)
6	13.89	149.05971 [M + H]^+^	131, 123, 103	Cinnamic acid (Chen et al. 2016)
7	15.15	133.06502 [M + H]^+^	115, 105, 103, 91, 79, 77, 55	Cinnamaldehyde (Chen et al. 2016)
8	18.08	163.07555 [M + H]^+^	145, 135, 121, 105, 79, 55	4-Methoxycinnamaldehyde (Chen et al. 2016)
9	28.84	239.10709 [M + H]^+^	238, 193, 131, 103, 91, 77, 65	Benzyl cinnamate (Son et al. 2014)

### Molecular docking study

**Figure 12.**
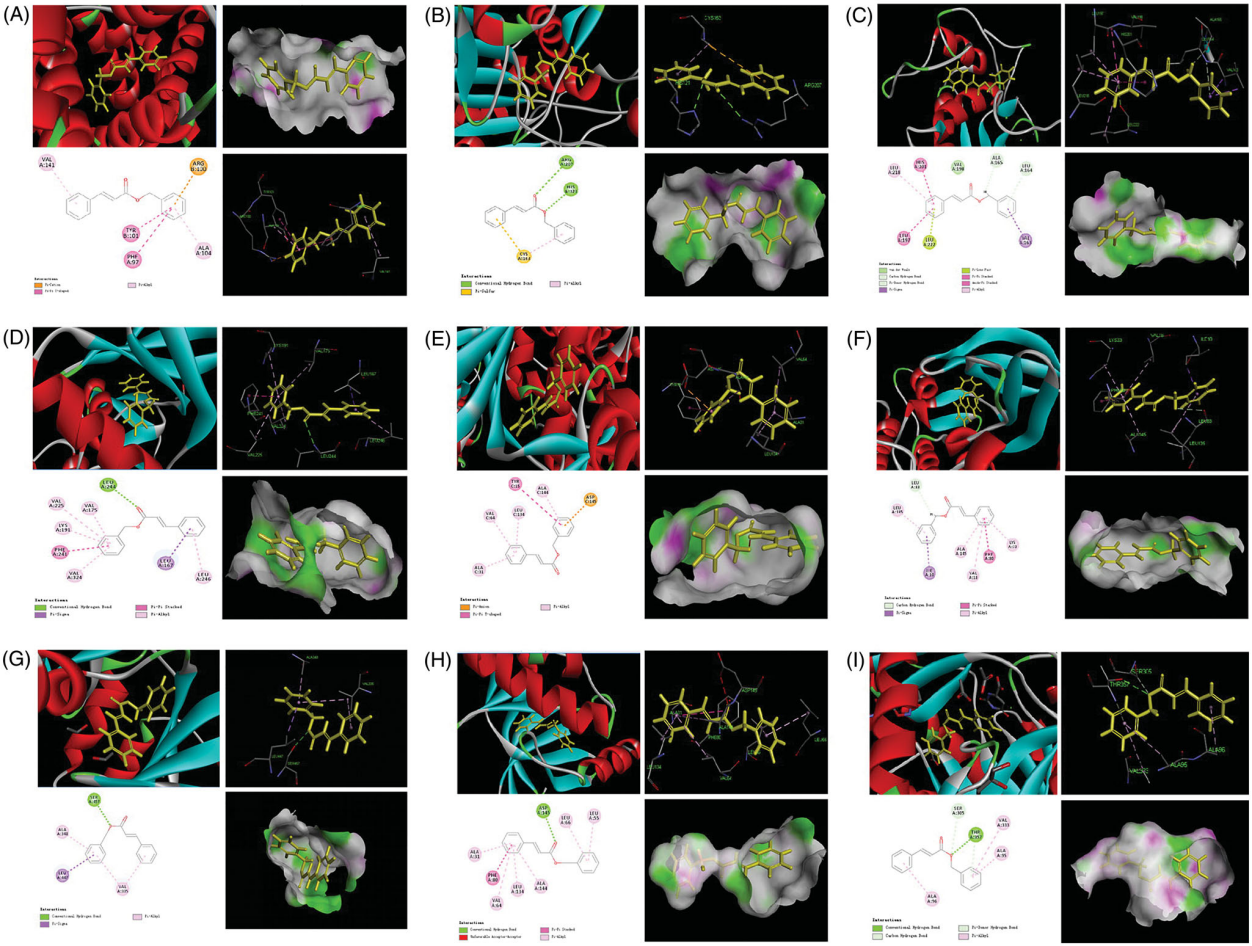
The represented results for the proposed action mode of molecular docking. Molecular docking analyses of benzyl cinnamate (compound **9**) to the binding site of human and Bcl-2 (A), caspase 3 (B), MMP3 (C), CDC2 (D), cyclin D (E), cyclin B1 (F), P21 (G), CDK4 (H) and P53 (I) proteins.

In order to study the active substance basis of *Cinnamomi ramulus* for its anti-RA effects, the molecular docking strategy was carried out to screen the identified 9 compounds. The active targets of molecular docking were proteins and genes studied in cell apoptosis, cell cycle, cell migration and invasion experiments. Nine potential protein targets were ultimately determined and their target protein–ligand crystal complexes were downloaded from the PDB (Table 3). The experimental results showed that compound **9** (benzyl cinnamate) had a good affinity with these selected protein targets, and its docking score was much higher than that of the prototype ligand of the target proteins, suggesting that compound **9** might be an important active component of *Cinnamomi ramulus* for its anti-RA effects. The results are shown in Figure 12.

**Table 3.** Information of the 9 potential target proteins investigated in the present study.

Uniport ID	PDB ID	Target Protein	Gene
P10415	6RNU	Apoptosis regulator Bcl-2	Bcl-2
P42574	3H0E	Caspase-3	Caspase 3
P14635	4Y72	G2/mitotic-specific cyclin-B1	Cyclin B1
P24385	6GUB	G1/S-specific cyclin-D1	Cyclin D
P38936	5XVA	Cyclin-dependent kinase inhibitor 1	P21
P04637	6ET4	Cellular tumour antigen p53	P53
P11802	1H00	Cyclin-dependent kinase 4	CDK 4
P06493	6FT8	Cyclin-dependent kinase 1	CDC 2
A5GZ70	1D5J	Matrix metalloproteinase 3	MMP3

### Benzyl cinnamate inhabits cell apoptosis and migration in synovial fibroblasts

**Figure 13.**
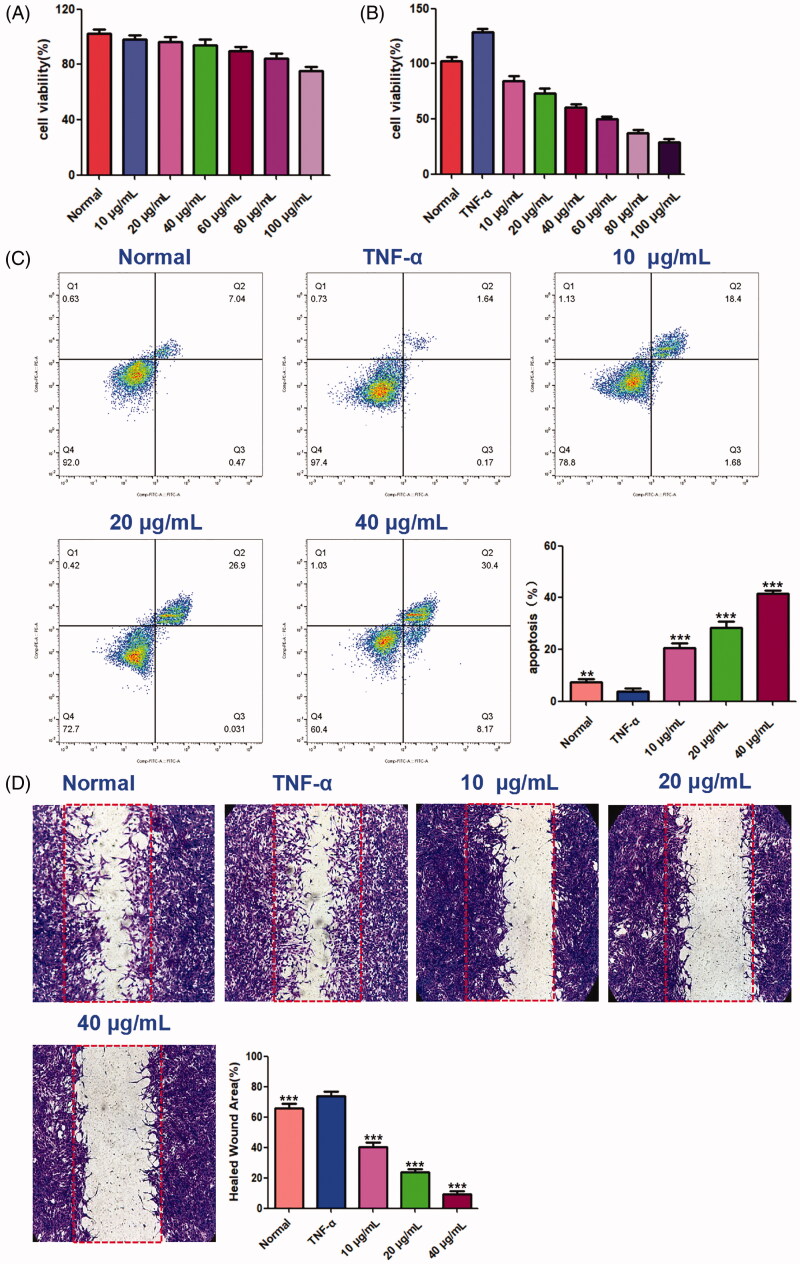
The effects of benzyl cinnamate on the proliferation, cell migration and apoptosis of MH7A cells. (A) HFLS and (B) MH7A cells were treated with different concentrations of benzyl cinnamate (10-100 µg/mL) for 24 h for the CCK-8 assays; (C) MH7A cells were treated with different concentrations of benzyl cinnamate (10, 20, 40 µg/mL) for 24 h, and the apoptotic cells were detected by Annexin V/PI assays; (D) MH7A cells were incubated with benzyl cinnamate (10, 20, 40 µg/mL) for 24 h for the cell migration assay. Data are expressed as mean ± SD (n = 3), **p < 0.01, ***p < 0.001, vs. TNF-α group.

The effect of benzyl cinnamate on MH7A cells viability was evaluated. Our results showed that benzyl cinnamate (10–100 µg/mL) did not affect cell viability of HFLS, while markedly reduced MH7A cells proliferation (Figure 13(A,B)). We chose 10, 20 and 40 μg/mL benzyl cinnamate for use in the follow-up experiments to reveal cell apoptosis and migration effects. As shown in Figure 13(C), the early and late apoptotic cells were both markedly increased after treatment with benzyl cinnamate. Moreover, the apoptosis rate increased with the increase of benzyl cinnamate concentration. As shown in Figure 13(D), cell migration was increased in MH7A cells treated with TNF-α alone compared with normal group, but benzyl cinnamate treatment markedly inhibited TNF-α-induced cell migration.

## Discussion

At present, the pathogenesis of RA is considered to be a multifactorial interaction process, and its occurrence and expression are influenced by many risk factors such as heredity and environment (Alamanos and Drosos 2005). The pathogenesis of RA is associated with a variety of cell types, and SFs has been identified as the responsible cell for destruction of cartilage and bone. Activated SFs in RA show excessive proliferation, loss of contact inhibition and increased migration (Bartok and Firestein 2010). Unfortunately, although there are many RA treatment options available, including traditional disease-modifying antirheumatic drugs (DMARDs) and currently available biologicals agents, the general effectiveness of these drugs has been far from satisfaction (Gabay et al. 2014). Therefore, we should pay more attention to the development of novel drugs for treating RA. Numerous previous reports indicated that the extract of *Cinnamomi ramulus* has a variety of biological actions, including anti-microbial, anti-inflammation, and anti-RA activities. On the basis of the known anti-inflammatory and anti-RA effects of *Cinnamomi ramulus*, we investigated the effects of ACR on RA using human synovial cell line MH7A cells and its underlying mechanisms.

In the present study, CCK-8 assays results showed that ACR significantly inhibited the cell proliferation in a dose-dependent manner, indicating that ACR exerted potent anti-proliferative effect on MH7A cells. To investigate the mechanism of ACR inhibiting synovial cell proliferation, we evaluated the ability of ACR to induce MH7A cells apoptosis and cycle arrest. Promoting programmed cell death (apoptosis) is an important strategy for RA therapy (Liu and Pope 2003; Li and Wan 2013; Zhang, Liu, et al. 2019). There are two main ways for apoptosis: the death receptor way and the intrinsic mitochondrial way. The intrinsic mitochondrial mediated apoptosis is considered to be the more critical way of the two (Stevens and Oltean 2019; Yoon et al. 2020). Mitochondrial mediated apoptosis is largely regulated by the Bcl-2 protein family. It is well known that among the Bcl-2 family proteins, anti-apoptotic proteins such as Bcl-2 can inhibit apoptosis, while pro-apoptotic proteins such as Bax can activate apoptosis (Wang and Zhao 2019). In this study, our results showed that ACR increased the expression of Bax in MH7A cells, as well as decreased the expression of Bcl-2 protein. Moreover, the expression of C-caspases-3 was significantly up-regulated in MH7A cells by ACR. These results collectively indicated that ACR has obvious pro-apoptotic effect on MH7A cells.

Cell cycle control is the major regulatory mechanism of cell growth, and the core of this process is the cyclin-dependent kinases (Cdks), which complex with the cyclin proteins (Dalton 2015). In G2-M transition, the cyclin-dependent protein kinase complex (CDC2-cyclin B1 complex) could be used as a marker of G2/M phase arrest (Park et al. 2000). P53 protein is a key tumour suppressor in cells. Through downregulating the expression of gene products critical to cell cycle progression, the activation of P53 tumour suppressor could lead to cell cycle arrest (Engeland 2018). Furthermore, P21 act as cyclin-dependent kinase inhibitors to arrest cells in the G2/M phase (Li et al. 2015). The cyclin D-CDK4 complex plays a role in G1 phase, and the cell cycle enters G1 phase when CDK4 and CDK6 form active complexes with D-type cyclins. However, a recent study found that CDK4 plays an unexpected role in the G2/M checkpoint (Brookes et al. 2015; Sheppard et al. 2015). In this study, we detected the distribution of cell cycle of MH7A cells by flow cytometry and found that ACR can significantly increase the proportion of MH7A cells in G2/M phase in a concentration-dependent manner. Therefore, we speculated that ACR could induce G2/M phase arrest of MH7A cells. To further verify this hypothesis, western blotting and q-PCR assays were performed to detect the expression levels of cycle-related proteins. The results showed that ACR could reduce the expression of CDC2 and cyclin B1 in MH7A cells and up-regulate P53, P21 and cyclin D. In addition, we also found that ACR can down-regulate the expression of CDK4. Therefore, ACR-induced G2/M blockade may be due to up-regulation of P53, P21 and cyclin D, and down-regulation of cyclin B1, CDC2 and CDK4.

Current studies have found that stimulated SFs migrated to intra-articular structures, leading to cartilage and bone damage, which is a major change in the pathogenesis of RA (Bottini and Firestein 2013). In the present study, the reduced numbers of transmembrane cells in transwell chambers and the scratch wound assays showed that ACR could suppress the migration and invasive capacity of MH7A cells. MMPs, a family of zinc-dependent proteases, are the main proteases for invasion and degradation of basement membranes and extracellular matrix. According to previous study, inhibition of MMPs can significantly reduce cell invasion and migration of synovial fibroblasts (Ma et al. 2014). In our study, we found out that ACR concentration-dependently suppressed the expressions of MMP-1, MMP-2 and MMP-3.

TCM has the characteristics of multiple components and multiple targets, resulting in its unclear basis of medicinal substances and unclear mechanism of action, which seriously restricts the development and promotion of TCM. In recent years, molecular docking method has become an important technology in the field of computer-aided drug research, which can realize rapid and high-throughput screening of potential drugs and greatly reduce the research and development cost and time (Peng et al. 2018). Therefore, more and more researchers have introduced molecular docking into the study of TCM. In this study, molecular docking results showed that the compounds in ACR had a good affinity with protein crystals. Notably, the docking fraction of benzyl cinnamate with nine proteins was higher than that of the prototype ligand, indicating that benzyl cinnamate may be closely related to the anti-RA effect of *Cinnamomi ramulus.* Previous studies have reported that benzyl cinnamate has anti-hypertension, antibacterial and antioxidant activities, etc. (Ohno et al. 2008; Tanapichatsakul et al. 2018). However, there are few reports about the effects of benzyl cinnamate on RA treatment. In this paper, we found that benzyl cinnamate induced early and late apoptosis and inhibited migration of MH7A cells in a dose-dependent manner. It could provide new clues for further study on the molecular mechanism of benzyl cinnamate in the treatment of RA.

## Conclusions

Taken together, this study suggested that *Cinnamomi ramulus* might be beneficial for relieving RA clinic symptoms through inhibiting proliferation, migration and invasion of SFs. Our results provide a basis for elucidating the molecular mechanisms of *Cinnamomi ramulus* on the therapeutic treatment of RA. Nevertheless, some limitations exist in our study. In order to better elucidate the characterization of *Cinnamomi ramulus* and its active components in RA, it will be further explored *in vivo* models in future studies.

## Supplementary Material

Supplementary_Figure_S1.tif
