# Supplementary figures and images for: Anti-proliferation and anti-migration effects of an aqueous extract of Cinnamomi ramulus on MH7A rheumatoid arthritis-derived fibroblast-like synoviocytes through induction of apoptosis, cell arrest and suppression of matrix metalloproteinase
Source: Pharm Biol. 2020 Sep 2;58(1):863–77. doi: 10.1080/13880209.2020.1810287 (PMC8641682; doi:10.1080/13880209.2020.1810287)

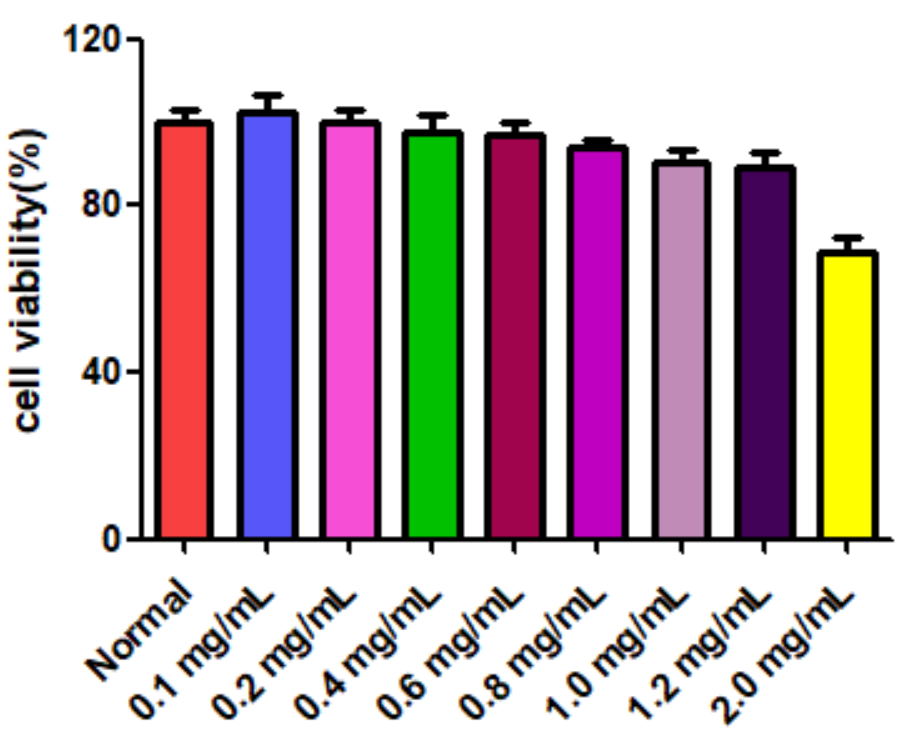

Supplement: Supplementary_Figure_S1.tif [file IPHB_A_1810287_SM4661.tif]
